# Division of the Portio-Dura; Excessive Pain

**Published:** 1844-08-10

**Authors:** 


### DIVISION OF THE PORTIO-DURA; EXCESSIVE PAIN.

The following brief narration is interesting in a physiological point of view. A dancing-master had a tumor developed over the articular surface of the lower joy, to remove which means of every kind were tried in vain: the tumor at length having acquired the size of half an apple, the patient consented to have it removed by operation, in the course of which, the surgeon saw that the trunk of the facial nerve passed right through the middle of the morbid mass, a mixture of steatoma and hydatids. Seeing no means of sparing it, the surgeon cut it through at one lusty stroke of the knife. The pain occasioned by this seemed horrible. The patient threw off the three assistants who were holding him, sprung from the seat, looked wildly around, and stretched out his hands in agony. The spectacle was made the more piteous by the semiparalysis of the face and distortion of the features that instantly ensued: the mouth was drawn completely over to the other side, the angle on the paralysed side hung down relaxed, the cheek lay hollow and meaningless; the eyes seemed sunken and smaller. It was by and by found that three-fourths of an inch of the trunk of the facial had been removed.

What is still farther remarkable is this: that the deformity did not continue even in the evening of the same day; the features had recovered themselves greatly, and after a fortnight, unless when the patient spoke, particular attention would have been required to perceive that anything was amiss with him.—*Bredow, in Caspar's Wochenschrift.—London Med. Gaz.*

